# Supplementation with Iron in Pulmonary Arterial Hypertension. Two Randomized Crossover Trials

**DOI:** 10.1513/AnnalsATS.202009-1131OC

**Published:** 2021-06-01

**Authors:** Luke S. G. E. Howard, Jianguo He, Geoffrey M. J. Watson, Li Huang, John Wharton, Qin Luo, David G. Kiely, Robin Condliffe, Joanna Pepke-Zaba, Nicholas W. Morrell, Karen K. Sheares, Anna Ulrich, Ruilin Quan, Zhihui Zhao, Xiaoli Jing, Chenhong An, Zhihong Liu, Changming Xiong, Peter A. Robbins, Timothy Dawes, Antonio de Marvao, Christopher J. Rhodes, Manuel J. Richter, Henning Gall, Hossein A. Ghofrani, Lan Zhao, Les Huson, Martin R. Wilkins

**Affiliations:** ^1^ National Heart and Lung Institute and; ^8^ Medical Research Council London Institute of Medical Sciences, Imperial College London, London, United Kingdom; ^2^ National Pulmonary Hypertension Service, Imperial College Healthcare National Health Service Trust, Hammersmith Hospital, London, United Kingdom; ^3^ Center of Pulmonary Vascular Disease, State Key Laboratory of Cardiovascular Disease, Fuwai Hospital, National Center for Cardiovascular Diseases, Chinese Academy of Medical Sciences and Peking Union Medical College, Beijing, China; ^4^ Department of Infection, Immunity and Cardiovascular Disease, University of Sheffield, Sheffield, United Kingdom; ^5^ National Pulmonary Hypertension Service, Sheffield Pulmonary Vascular Unit, Royal Hallamshire Hospital, Sheffield, United Kingdom; ^6^ National Pulmonary Hypertension Service, Royal Papworth Hospital, Cambridge, United Kingdom; ^7^ Department of Physiology, Anatomy and Genetics, University of Oxford, Oxford, United Kingdom; ^9^ Department of Internal Medicine, Justus-Liebig-University Giessen, Universities of Giessen and Marburg Lung Center, Member of the German Center for Lung Research (Deutsches Zentrum für Lungenforschung), Giessen, Germany; and; ^10^ Department of Pneumology, Kerckhoff Heart, Rheuma and Thoracic Center, Bad Nauheim, Germany

**Keywords:** pulmonary arterial hypertension, iron, ferric carboxymaltose, iron dextran, exercise capacity

## Abstract

**Rationale:** Iron deficiency, in the absence of anemia, is common in patients with idiopathic and heritable pulmonary arterial hypertension (PAH) and is associated with a worse clinical outcome. Oral iron absorption may be impeded by elevated circulating hepcidin concentrations. The safety and benefit of parenteral iron replacement in this patient population is unclear.

**Objectives:** To evaluate the safety and efficacy of parenteral iron replacement in PAH.

**Methods:** In two randomized, double-blind, placebo-controlled 12-week crossover studies, 39 patients in Europe received a single infusion of ferric carboxymaltose (Ferinject) (1,000 mg or 15 mg/kg if weight <66.7 kg) or saline as placebo, and 17 patients in China received iron dextran (Cosmofer) (20 mg iron/kg body weight) or saline placebo. All patients had idiopathic or heritable PAH and iron deficiency at entry as defined by a serum ferritin <37 μg/L or iron <10.3 μmol/L or transferrin saturations <16.4%.

**Results:** Both iron treatments were well tolerated and improved iron status. Analyzed separately and combined, there was no effect on any measure of exercise capacity (using cardiopulmonary exercise testing or 6-minute walk test) or cardiopulmonary hemodynamics, as assessed by right heart catheterization, cardiac magnetic resonance, or plasma NT-proBNP (N-terminal–pro hormone brain natriuretic peptide) at 12 weeks.

**Conclusions:** Iron repletion by administration of a slow-release iron preparation as a single infusion to patients with PAH with iron deficiency without overt anemia was well tolerated but provided no significant clinical benefit at 12 weeks.

Clinical trial registered with ClinicalTrials.gov (NCT01447628).

Pulmonary arterial hypertension (PAH) arises from increased pulmonary vascular resistance (PVR) and predisposes to right heart failure and premature death. Idiopathic PAH, a diagnosis by exclusion, is commonly associated with iron deficiency (1–4). Iron deficiency, in the absence of overt anemia, is in turn associated with worse functional capacity and survival in PAH. For example, circulating sTfR (soluble transferrin receptor) concentrations, a marker of iron status largely unaffected by inflammation, >28.1 nmol/L are associated with twice the risk of a premature death (1). The European Respiratory Society and European Cardiac Society guidelines for the management of pulmonary hypertension suggest that iron replacement should be considered as part of the treatment plan for these patients (5). However, there remain concerns about the safety of iron supplementation in idiopathic and heritable PAH, and its impact on PVR, cardiac function, and exercise capacity remains to be defined.

One open-label study of five patients with PAH has reported an increase in 6-minute walk distance 16 weeks after oral iron supplementation (6). Oral iron is relatively ineffective at correcting serum iron concentrations in PAH (2), at least in part because circulating hepcidin, which inhibits iron absorption from the gut, is inappropriately raised in patients with iron deficiency and PAH (1). A recent open-label study with oral ferric maltol in 20 patients with pulmonary hypertension of mixed etiology and iron deficiency anemia showed an increase in iron and hemoglobin concentrations at 12 weeks, together with an improvement in 6-minute walk distance and signs of right ventricular function (7). Intravenous iron administration is more effective at restoring iron, and two small open-label studies have reported improvements in exercise capacity and quality of life after single infusions of high-dose ferric carboxymaltose (Ferinject) (8, 9).

Here, we report two randomized, double-blind studies of intravenous iron versus placebo, analyzed separately and combined, which have investigated the safety and efficacy of iron replacement in patients with idiopathic or heritable PAH who have iron deficiency without anemia. A discussion describing the rational for the study and its design has been published previously (10).

## Methodology

### Patients

Patients with idiopathic or heritable PAH and iron deficiency and who had been stable on their current therapy for the preceding 1 month were recruited. PAH was defined by a resting mean pulmonary artery pressure ≥25 mm Hg, pulmonary artery wedge pressure ≤15 mm Hg, and normal or reduced cardiac output on right heart catheterization (5). Iron deficiency was defined by sTfR concentrations >28.1 nmol/L. Because this assay was not generally accessible outside the research setting, we derived cutoff criteria from readily available laboratory tests to predict sTfR >28.1 nmol/L (data not shown), with patients requiring one of the following: ferritin <37 μg/L or iron <10.3 μmol/L or transferrin saturations <16.4% (10). These criteria were derived from profiling data from a cohort study in the U.K. population (1) and were estimated to predict correct classification of patients with sTfR >28.1 nmol/L, with 83% true positives. Patient randomization was stratified by sex with an appropriate fixed block size to ensure equal allocation to iron or placebo, and data were collected using an InForm database. Data entry in Europe was monitored by Dr. Oestreich and Partner, Koln. Harmonization of protocols and data entry in China was reviewed on location in Beijing before unblinding. The studies were registered under clinicaltrials.gov NCT01447628 with EudraCT Number 2010–024585–22.

### Study 1

Thirty-nine patients recruited from four specialist centers in Europe (Hammersmith Hospital, London; Papworth Hospital, Cambridge; Royal Hallamshire Hospital, Sheffield; and Universities of Giessen and Marburg Lung Center, Germany) between March 2012 and July 2017 were randomized 1:1 to a single infusion of 1,000 mg (or 15 mg/kg if weight <66.7 kg) Ferinject or saline given over 15 minutes, with crossover after 12 weeks (Figure E1a) (10). Patients were recruited by local investigators, and all gave their informed consent. The infusions were prepared by the local pharmacy and delivered in an opaque infusion set with line covers by an unblinded team, who were separate from the trial management and delivery team, to maintain blinding for the patient. The patient was blindfolded while the line was connected, and the arm was covered during the procedure. All investigators responsible for patient recruitment and care and for data acquisition and analysis were blind to treatment assignment. The data were unblinded by the statistician for the study.

The original null hypothesis was that iron replacement has no effect on PVR in patients with iron deficiency and idiopathic or heritable PAH, with a two-sided alternative that iron replacement changes PVR in patients with iron deficiency and idiopathic PAH. Assuming an SD of 250 dynes/s/cm^5^ and a drop-out rate of 10%, 60 patients randomized 1:1 was estimated to give this study an 80% power to detect a 194-dynes/s/cm^5^ reduction in PVR with iron treatment at a significance of *P* = 0.05 using a two-sample *t* test (10). Patient recruitment was slow. An unplanned blinded analysis of the PVR endpoint after 15 patients indicated that the study had conditional power (under the current trend hypothesis) of <60% to detect a statistically significant effect. A published report from an open-label study suggesting an improvement in endurance time after Ferinject administration (9) prompted a blinded conditional power analysis based on the exercise data; this showed that recruiting an additional 30 patients would have a 90% power to detect a significant change in oxygen consumption (V̇o_2_) and >80% power for change in endurance time on cardiopulmonary exercise testing. A protocol amendment was made, changing the primary endpoint from PVR to change in endurance time on bicycle cardiopulmonary exercise test at 80% peak work rate (derived from the incremental test); cardiac catheterization was dropped from the protocol. The trial was stopped after 39 patients because of slow recruitment.

Predefined secondary endpoints for the study comprised changes from baseline in the following: peak V̇o_2_ (ml/min/kg), V̇o_2_ at metabolic threshold, minute ventilation (V̇e)/carbon dioxide production (V̇co_2_) slope, V̇o_2_/work rate slope, and O_2_ pulse derived from the incremental bicycle cardiopulmonary exercise test (11); V̇o_2_ at the end of the endurance bicycle cardiopulmonary exercise test at 80% peak work rate determined from the incremental exercise test; gas exchange at 3 minutes after the start of the work phase of an endurance exercise test at 80% peak work rate (determined from the incremental exercise test); serum iron, transferrin saturation, ferritin, and sTfR); 6-minute walk distance and Borg dyspnea scale (12); New York Heart Association (NYHA) World Health Organization functional class; NT-proBNP (N-terminal–pro hormone brain natriuretic peptide); right and left ventricular ejection fraction and volumes derived from cardiac magnetic resonance (MR). Quality of life was assessed using the Cambridge Pulmonary Hypertension Outcome Review, (13) and response to intervention was measured with the Patient Global Assessment (PGA).

All measurements of iron status (serum iron and ferritin and transferrin saturation) made by local hospitals at entry were repeated by a central laboratory (North West London Pathology Laboratory hosted by Imperial College Healthcare NHS Trust) and used to define iron deficiency in the study. NT-proBNP and hepcidin assays were also performed by the central laboratory. All cardiac MR analyses were completed by a single center (Hammersmith Hospital) using two independent observers who were blinded to the treatment received.

Approval for the study was given by the National Research Authority (11/LO/0095).

### Study 2

Seventeen patients were recruited from Fu Wai Hospital in Beijing between June 2015 and June 2017 (Figure E1b). The European protocol was adopted, but patients were randomized to a single infusion of 20 mg iron/kg body weight Cosmofer or saline administered over 4–6 hours, with crossover after 12 weeks. Patients were recruited by local investigators, and all gave informed consent. The treatment was prepared by a local independent pharmacist and delivered in the same fashion as study 1 to maintain blinding of the patient and research team.

The primary outcome measure was the change in resting PVR from baseline to 12 weeks, which was measured by cardiac catheterization, based on the same power calculation given for the Europe study. The predefined secondary endpoints in this study were change from baseline at 12 weeks in the following: peak V̇o_2_ (ml/min/kg), V̇o_2_ at metabolic threshold, V̇e/V̇co_2_ slope, V̇o_2_/work rate slope, and O_2_ pulse from an incremental bicycle cardiopulmonary exercise test; serum iron, transferrin saturation and ferritin; 6-minute walk distance and Borg dyspnea scale; NYHA World Health Organization functional class; and right and left ventricular ejection fraction and volumes derived from cardiac MR.

All cardiac MR analyses were completed by a single center (Hammersmith Hospital) using two independent observers who were blinded to the treatment received.

The study protocol was approved by the institutional review board of Fuwai Hospital (approval number 2014–601). The study was stopped after 17 patients because of difficulties in recruiting.

## Statistics and Data Analysis

### Intention-to-Treat Analysis

All primary and secondary efficacy endpoints (with the exception of the PGA and the NYHA classification) were analyzed using linear mixed models appropriate for a crossover design. These linear models included administration sequence, period, and treatment as fixed effects and included subject as a random effect, and all models included the baseline value of the endpoint as a covariate.

The PGA endpoint was analyzed using a Cumulative Link Mixed Model appropriate for the categorical nature of this endpoint. NYHA class was not formally analyzed because almost all patients remained in the same NYHA class throughout the study.

Residuals from the linear models were tested for normality using the Shapiro-Wilks test. Significant departure from normality was detected for some endpoints, and exploratory analysis with various possible data transformations did not correct this issue. For all endpoints, whether residuals appeared to be normally distributed or not, a nonparametric *P* value testing the treatment effect was also derived using a permutation test. Normal theory and nonparametric *P* values were very similar in all cases.

From the linear mixed models, estimates of the treatment effect (difference in mean outcomes between the two treatment arms) were derived. These were summarized using adjusted (least-squares) means and mean differences, together with the SEM difference and associated *P* values. *P* values testing for sequence and period effects were also derived for each of the linear mixed models.

All analyses were conducted using the intention-to-treat (ITT) analysis sets (United Kingdom: *N* = 39; China: *N* = 17). Missing data values were imputed using a predictive-means multiple imputation algorithm, and results were derived across 100 multiple imputations.

### *Post Hoc* Per-Protocol Analysis

One patient in the Europe study received placebo twice and was included in the ITT. A per-protocol analysis was conducted in the Europe study to explore the relationship between change in outcome and change in iron status, defined by the change in log ratio of sTfR and ferritin (log sTfR/ferritin).

## Results

The baseline characteristics of the patients recruited to both studies are shown in Table 1.

**Table 1.** Baseline characteristics of patients recruited from Europe and China

Characteristic	Europe	China
Sex, F:M, *n*	29:10	15:2
Age, yr	49 (14.5)	30 (11.0)
BMI, kg/m^2^	27.2 (6.0)	23.3 (4.2)
Chinese, *n*	—	17
White, *n*	37	—
South Asian, *n*	2	—
Iron, μmol/L	12.2 (10.2)	9.0 (6.0)
Ferritin, μg/L	17.0 (21.8)	11.0 (7.0)
sTfR, nmol/L	39.1 (24.4)	—
Hepcidin, ng/ml	1.4 (4.1)	—
Hemoglobin, g/dl	13.7 (2.7)	13.0 (2.9)
NYHA functional class, *n*		
I	1	1
II	20	12
III	18	4
6MWD, m	427.0 (85.0)	462.0 (118.0)
Dyspnea score (Borg index)	2.8 (2.0)	1.0 (2.5)
NT-proBNP, ng/L	127.3 (335.8)	—
Systolic blood pressure, mm Hg	112.0 (16.5)	106.0 (10.0)
Diastolic blood pressure, mm Hg	68.0 (11.5)	70.0 (8.0)
PVR, Wood units	6.7 (3.9)	9.3 (3.3)
mPAP, mm Hg	47.0 (8.5)	49.0 (16.0)
mRAP, mm Hg	10.0 (7.5)	3.0 (6.0)
mPAWP, mm Hg	11.0 (2.8)	7.0 (5.0)
Cardiac index, L/min/m^2^	2.6 (1.2)	2.7 (0.7)
Treatment, *n*		
PDE-5 inhibitor	33	13
Endothelin receptor antagonist	29	7
Prostacyclin	10	1
Calcium channel blocker	2	—
Warfarin	26	9
Diuretics	24	17
Digoxin	4	2
Sodium ferulate	—	16

*Definition of abbreviations*: 6MWD = 6-minute walk distance; BMI = body mass index; mPAP = mean pulmonary arterial pressure; mPAWP = mean pulmonary artery wedge pressure; mRAP = mean right atrial pressure; NT-proBNP = N-terminal–pro hormone brain natriuretic peptide; NYHA = New York Heart Association; PDE-5 = phosphodiesterase type 5; PVR = pulmonary vascular resistance; sTfR = soluble transferrin receptor.

All continuous variables are median (interquartile range).

### Study 1

At entry, all 39 study participants from Europe met at least one criterion for iron deficiency (serum iron, ferritin, or transferrin saturation); 28 (74%) met the sTfR threshold >28.1 nmol/L. One patient who received placebo on two occasions was included in the ITT but not the per-protocol analysis. No one was lost to follow up.

**Figure 1.**
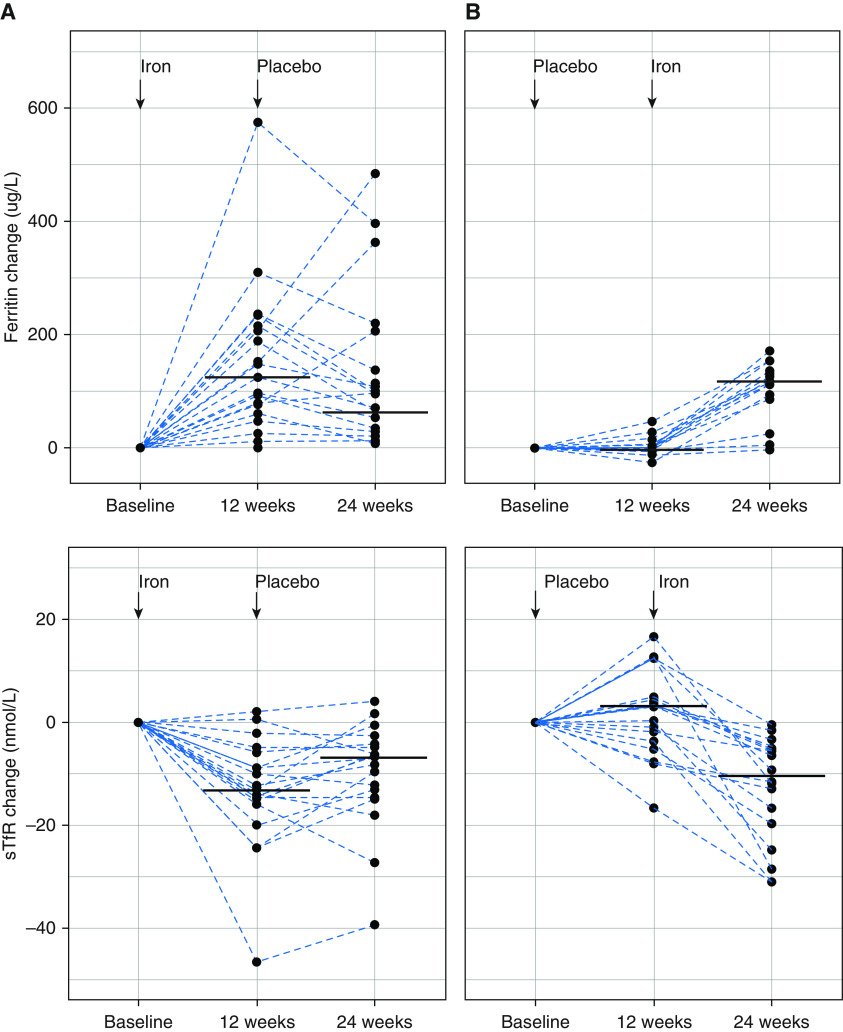
Change in ferritin and sTfR (soluble transferrin receptor) concentrations after infusion of Ferinject and placebo. Change in ferritin (top panels) and sTfR (bottom panels) concentrations, plotted individually (dotted lines) and as medians (black bars), from baseline to 12 and to 24 weeks respectively for patients who received either iron (*A*) or placebo (*B*) first.

Iron treatment led to an increase in ferritin (*P* = 0.0003; Table E1) from 17 μg/L at baseline to 146 μg/L at 12 weeks in the group that received iron first (iron_first_) and from 14 μg/L to 134.5 μg/L from 12 to 24 weeks in the group that received placebo first (placebo_first_) (Figure 1A and Table E2). The sTfR concentrations decreased in both groups (*P* < 0.0001; Table E1), consistent with improvement in iron status (Figure 1B and Table E2). More detailed pharmacokinetic analysis in 14 patients sampled at 2 weeks showed interindividual variability in the magnitude of response to iron, but the pattern was to decline after an early peak and to remain stable within the normal range from 12 weeks to beyond 24 weeks (Figure E2).

**Figure 2.**
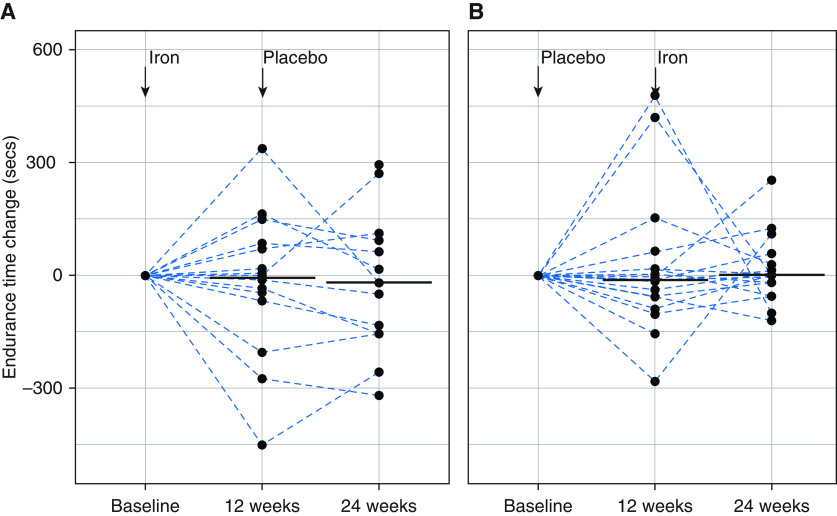
Change in endurance time after infusion of Ferinject. Change in endurance time, plotted individually (dotted lines) and as medians (black bars), from baseline to 12 and to 24 weeks respectively for patients who received either iron (*A*) or placebo (*B*) first.

Iron treatment had no significant effect on endurance time measured on cardiopulmonary exercise testing (Figure 2) or any of the secondary endpoints (Tables E1 and E2 and Figure E3).

The effect of change in iron status on response to iron infusion was examined using the log-transformed ratio of sTfR and ferritin (log sTfR/ferritin), which has been proposed as an optimal measure to distinguish between anemia of chronic disease and iron deficiency anemia (14). A decrease in log sTfR/ferritin signals improvement in iron availability. There was no significant change in any parameter measured in response to an improvement in iron status after correcting for multiple testing (Figure E4).

Ferinject was well tolerated, and no serious adverse events were reported. Phosphate concentrations, which can fall in the first 2 weeks after ferric carboxymaltose, remained in the normal range throughout (Figure E5).

### Study 2

All 17 patients recruited in China completed the crossover design. All participants were iron deficient at baseline as defined by at least one of serum iron concentrations, serum ferritin, or transferrin saturation (Table 1); 59% patients met all three biomarker cutoffs. sTfR concentrations were not measured in China. No one was lost to follow up.

**Figure 3.**
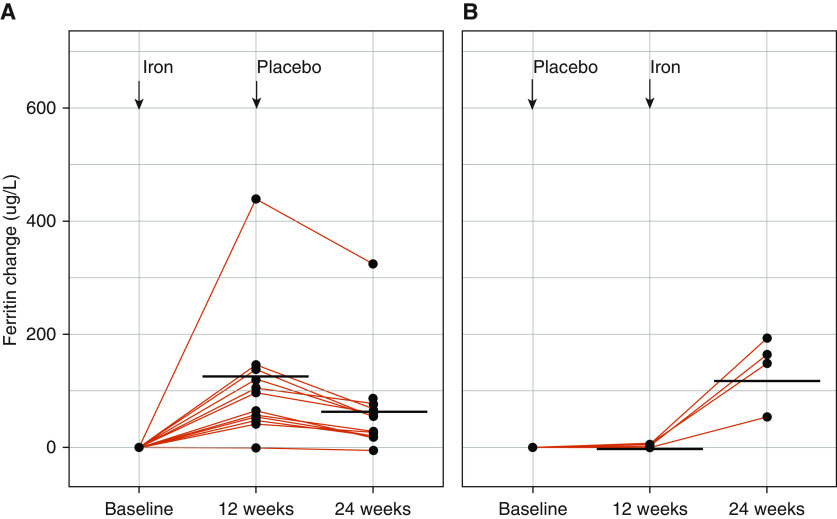
Change in ferritin after infusion of Cosmofer. Change in ferritin concentrations, plotted individually (red lines) and as median (black bar), from baseline to 12 and to 24 weeks respectively for patients who received either iron (*A*) or placebo (*B*) first.

**Figure 4.**
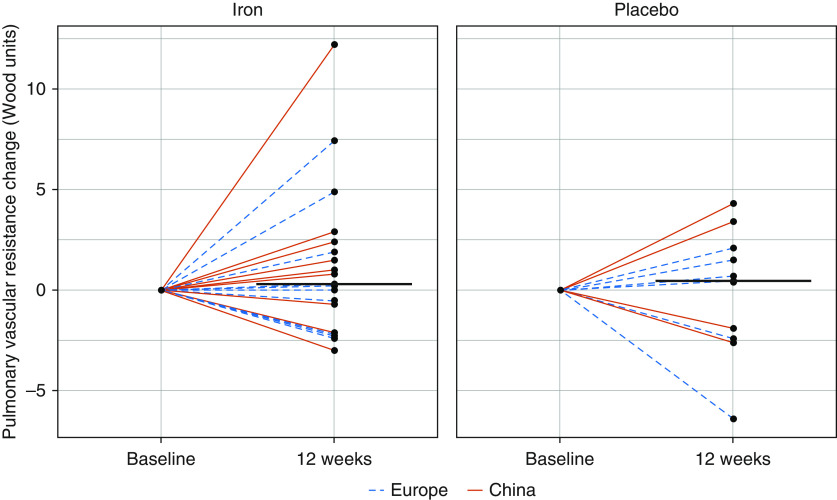
Change in pulmonary vascular resistance in combined Europe and China studies. Change in PVR, plotted individually (dotted blue lines indicate Europe; solid red lines indicate China) and as medians (black bars), for patients who received either iron or placebo first. PVR = pulmonary vascular resistance.

Treatment with Cosmofer increased serum ferritin concentrations (Figure 3 and Tables E3 and E4) but had no effect on the primary outcome (PVR) (Figure 4 and Tables E3 and E4). There was no significant effect on any secondary endpoint (Tables E3 and E4 and Figure E3). Cosmofer was well tolerated.

### Combined Analysis

Patient-level data from the two studies were pooled to investigate the effect of iron replacement on PVR (*n* = 28; Figure 4), 6-minute walk distance (*n* = 53), and peak V̇o_2_ (*n* = 54) and V̇o_2_ at metabolic threshold (*n* = 52) on incremental cardiopulmonary exercise testing (Figure E3). No beneficial signal was observed. Iron replacement had no significant effect on cardiac function (*n* = 37) at 12 weeks as measured by repeat cardiac MR (Table E5 and Figure E6).

## Discussion

Iron was administered as a single infusion in the form of Ferinject or Cosmofer to patients with idiopathic or heritable PAH and iron deficiency in a dose, adjusted to body weight, that was sufficient to improve their iron status. It was well tolerated but had no measurable effect on exercise capacity or PVR when analyzed separately or combined.

Iron deficiency can be difficult to define in chronic diseases, particularly those in which inflammation has a prominent role. Inflammation is associated with higher ferritin concentrations but lower serum iron and transferrin saturation. Circulating sTfR is largely unaffected by inflammation, and concentrations >28.1 nmol/L are associated with poor clinical outcomes in idiopathic and heritable PAH (1). Because sTfR assays are not routinely available, we recruited patients if they met one of the following three criteria: serum ferritin <37 μg/L, iron <10.3 μmol/L, and transferrin saturations <16.4% (10). In practice, 74% of patients recruited from Europe had an sTfR concentration >28.1 nmol/L at baseline. The log sTfR/ferritin ratio has been suggested to be a better measure of iron deficiency (14). An exploratory analysis of response to iron in this patient subgroup again showed no improvement in exercise capacity.

The pulmonary vascular pressor response to alveolar hypoxia is influenced by iron status; iron deficiency exaggerates, and iron replacement partially attenuates, this response (15–17). Two open-label studies of parenteral iron replacement in patients with idiopathic and heritable PAH and iron deficiency have reported that a single infusion of ferric carboxymaltose improves exercise capacity and quality of life. A study of 20 patients receiving 800–1,000 mg (mean 925 mg) iron recorded an improvement of 37.8 m in 6-minute walk distance at 8 weeks compared with a repeat measurement in a matched group stable on their existing treatment (8). A subset of eight patients underwent cardiopulmonary exercise testing, and an increase in anaerobic threshold, a marker of oxygen delivery/utilization during exercise, was observed. A study of 15 patients did not find any significant change in 6-minute walk test but did measure an increase in endurance time and aerobic capacity on cardiopulmonary exercise testing, both secondary endpoints, at 12 weeks (9). This could not be explained by improved cardiac function, but increased oxygen handling by quadriceps muscle cells was hypothesized to play a role.

Open-label studies have well-recognized limitations compared with randomized, double-blind, placebo-controlled studies, particularly when a subjective endpoint such as exercise capacity is used and when patients might perform better on repeat testing because of familiarity with the test. An interesting observation in our report is the exercise response of patients to repeat testing in the second half of the crossover design. Both the iron_first_ and placebo_first_ patients began iron deficient, but after 12 weeks, the iron_first_ patients were now iron replete as judged by their ferritin and sTfR concentrations. In contrast, the placebo_first_ patients remained iron deficient during their first 12 weeks and became iron replete during Weeks 12–24. Both groups would have undergone two previous exercise tests by Week 24, reducing the effect of training; the response in the placebo group between baseline and 12 weeks captures the variability in the measurement. Inspection of the endurance time achieved during the third test at 24 weeks shows no signal of improvement from iron administration.

These data are at odds with experience in chronic heart failure, in which iron deficiency is present in around 50% of patients (18). An early study randomized 35 patients with left heart failure to 16 weeks of intravenous iron (200 mg weekly until ferritin 500 ng/ml, 200 mg monthly thereafter) or no treatment in a 2:1 ratio (19). Intravenous iron loading improved exercise capacity and symptoms; the benefits were more evident in patients with anemia. A meta-analysis of four randomized controlled studies involving 839 patients (504 randomized to ferric carboxymaltose) has reported that patients receiving iron had lower rates of recurrent cardiovascular hospitalizations and mortality (20). In contrast to these studies in PAH, patients recruited to heart failure studies are more frequently anemic (45% receiving iron in the meta-analysis), with a higher proportion in functional class III (20), and so perhaps more likely to benefit from iron replacement.

The prevalence of iron deficiency in idiopathic and heritable PAH has led to interest in whether it is a causative factor in the pathogenesis of this condition. In support of an etiological role, chronic severe iron deficiency in rats leads to pulmonary hypertension with pulmonary vascular remodeling, which is reversible with iron replacement (21). A mouse model of intracellular iron deficiency achieved by targeted knock-in of a hepcidin-resistant isoform of the iron export protein ferroportin develops pulmonary hypertension, which can be prevented and reversed by intravenous iron (22). Manipulating iron availability influences the pressor response to hypoxia in humans, a response driven by hypoxia-induced pulmonary vasoconstriction. Contrary to these arguments are the findings from a recent study using two-sample Mendelian randomization (23). Mendelian randomization uses genetic variants as instruments to investigate causal relationships between risk factors (here, iron deficiency) and phenotype (PAH). The study compared the distribution of genetic variants that influence red cell distribution width, a marker of iron deficiency, in four genotyped PAH cohorts with a control population. Overrepresentation of variants associated with elevated red cell distribution width (a surrogate for low iron concentrations) in PAH would imply a causative link. The study reported no evidence for a mechanistic link between iron deficiency and PAH (odds ratio, 1.07; 95% confidence interval, 0.92–1.24).

Although iron deficiency is prevalent in PAH, patient recruitment proved a challenge in these studies. The requirement for more than one cardiac catheter was not popular, but even so, recruitment was slow, a reason why commercial studies with pressurized timelines engage multiple centers to recruit. The subjects recruited in China were younger than in Europe and in both studies were mainly in functional class II, which may reduce the sensitivity to detect therapeutic benefit. Another factor possibly limiting sensitivity is that the patients studied had significant but moderate iron deficiency; their sTfR concentrations have been shown to be associated with poor outcomes (1), but their hemoglobin concentrations were within the normal range. It is possible that an assessment early after iron infusion might have detected a signal of benefit in exercise function, but, although different parenteral iron preparations were used in the two studies for licensing reasons, both increased iron stores at 3 months compared with baseline. Indeed, there was a pharmacological carryover effect with iron status improved out to 24 weeks but no carry over of pharmacodynamic effect, as none was observed.

In summary, iron deficiency is common in idiopathic and heritable PAH and can be safely managed by a single intravenous administration of a slow-release iron preparation, adjusted for body weight. Iron replacement in the absence of overt anemia has no clinically significant impact on markers of disease severity or quality of life at 12 weeks. Patients with iron deficiency should not be denied iron replacement at the discretion of the physician, but this report should temper the expectation of therapeutic benefit from iron replacement in patients with PAH and iron deficiency in the absence of anemia.
